# The sesquiterpene alcohol farnesol mitigates cadmium hepatotoxicity by attenuating oxidative stress and NF-kappaB/NLRP3 inflammasome axis and upregulating PPARgamma in rats

**DOI:** 10.17179/excli2024-7488

**Published:** 2024-11-12

**Authors:** Reem S. Alruhaimi, Emad H.M. Hassanein, Sulaiman M. Alnasser, Mohammed A. Alzoghaibi, Omnia A.M. Abd El-Ghafar, Mostafa K. Mohammad, Ibrahim Elbagory, Ayman M. Mahmoud

**Affiliations:** 1 Department of Biology, College of Science, Princess Nourah bint Abdulrahman University, Riyadh 11671, Saudi Arabia; 2 Department of Pharmacology & Toxicology, Faculty of Pharmacy, Al-Azhar University-Assiut Branch, Assiut 71524, Egypt; 3 Department of Pharmacology and Toxicology, College of Pharmacy, Qassim University, Qassim 51452, Saudi Arabia; 4 Physiology Department, College of Medicine, King Saud University, Riyadh, 11461, Saudi Arabia; 5 Department of Pharmacology and Toxicology, Faculty of Pharmacy, Nahda University, Beni-Suef 62764, Egypt; 6 Department of Pharmacology and Toxicology, Faculty of Pharmacy, Badr University in Assiut, New Nasser City, West of Assiut, Assiut 71523, Egypt; 7 Department of Pharmaceutics, Faculty of Pharmacy, Northern Border University, Rafha 76321, Saudi Arabia; 8 Department of Life Sciences, Faculty of Science and Engineering, Manchester Metropolitan University, Manchester M1 5GD, UK; 9 Molecular Physiology Division, Zoology Department, Faculty of Science, Beni-Suef University, Beni-Suef 62514, Egypt

**Keywords:** heavy metals, hepatotoxicity, oxidative stress, inflammation, farnesol

## Abstract

Farnesol (FAR) is a sesquiterpene alcohol that exists in many fruits and vegetables and possesses promising anti-inflammatory and antioxidant activities. Cadmium (Cd) is an environmental pollutant known for its serious health effects. Liver injury associated with oxidative stress is a hazardous consequence of exposure to Cd. This study evaluated the effect of FAR on Cd-induced oxidative stress, inflammation, and hepatocyte injury, pinpointing the involvement of NF-κB/NLRP3 inflammasome axis, TGF-β/Smad3 signaling and PPARγ. FAR was supplemented for 14 days and rats received Cd on day 7. Elevated serum transaminases, ALP and LDH, decreased albumin, and multiple histopathological alterations were observed in Cd-administered rats. Cd increased liver MDA and NO, decreased GSH and antioxidant enzymes, and upregulated NF-κB p65, IL-6, TNF-α, iNOS, NLRP3, ASC, caspase-1, IL-1β, and cleaved caspase-3. TGF-β, Smad3 phosphorylation and α-SMA were upregulated, and collagen deposition was increased in Cd-administered rats. FAR ameliorated liver injury markers and tissue alterations, attenuated oxidative stress, suppressed NF-κB/NLRP3 inflammasome axis and TGF-β/Smad3 signaling, and enhanced antioxidants. In addition, FAR downregulated caspase-3 and pro-inflammatory cytokines and increased liver PPARγ in Cd-administered rats. *In silico*, FAR showed affinity to bind ASC and NLRP3 PYD domains, TGF-β, and PPARγ. In conclusion, FAR protects the liver against Cd toxicity by suppressing oxidative stress, inflammatory response and cell death, effects linked to modulation of NF-κB/NLRP3 inflammasome axis, TGF-β/Smad3 signaling and PPARγ.

## Introduction

Heavy metals (HMs) are hazardous environmental pollutants that can cause serious health problems in the main organs. HMs can accumulate in the body because of their non-biodegradable nature and this accumulation can negatively impact the cell structure and function (Renu et al., 2021[51]). Cadmium (Cd) has been the subject of international concern since it can directly or indirectly endanger human health. Cd is a HM environmental toxicant that can reach the body through cigarette smoke, air pollution, water, or from activities of industries such as mining, welding, or oil extraction (Järup and Åkesson, 2009[25]; Friberg et al., 2019[18]). Furthermore, the development of Cd-containing nanomaterials used in different applications could also be a source of Cd intoxication (Rzigalinski and Strobl, 2009[53]). The risk of exposure is increased since Cd can easily enter the food chain and impairs different organ systems (Oskarsson et al., 2004[45]). Exposure to Cd can result in serious disorders, including bone, renal, cardiovascular, neurological, and hepatic dysfunctions (Satarug et al., 2010[57]). In addition to its direct cytotoxic effects on different tissues, Cd has been classified as a category Ⅰ human carcinogen (IARC, 1993[22]). There are no specific transport channels for the Cd to enter the cell, but it has similar physical and chemical properties to other essential metals (Vesey, 2010[65]). Cd toxicity is linked to induction of oxidative stress (OS) and impairment of cellular defence mechanism (Lin et al., 2007[37]; Liu et al., 2009[39]). Cd indirectly provokes the generation of reactive oxygen species (ROS), such as superoxide (• O_2_) and hydroxyl (• OH) radicals, and hydrogen peroxide (H_2_O_2_). The generated ROS can react with cellular biomolecules, resulting in lipid peroxidation (LPO), DNA damage, protein malfunction, altered gene expression, and cellular apoptosis (Casalino et al., 1997[9]; Ikediobi et al., 2004[23]; Cuypers et al., 2010[12]). In addition, Cd is implicated in the depletion of glutathione (GSH) and other essential endogenous antioxidants which contribute markedly to OS and apoptosis. Although any organ could be affected by Cd, the liver and kidneys are the most sensitive tissues to Cd intoxication due to their ability to synthesize metallothionein (MT); a protein that scavenges and reduces Cd accumulation within the tissues after ingestion (Wolff et al., 2006[68]; Abouhamed et al., 2007[1]). Once Cd is absorbed by pulmonary or intestinal cells, it enters the systemic circulation and is finally deposited in different organ tissues, mainly liver and kidneys (Egger et al., 2019[17]; Young et al., 2019[70]). The vast majority of the absorbed amount of Cd by gastrointestinal tract is transported to hepatocytes via portal circulation. The liver is the most important tissue that can detect cellular injury and OS, as it is considered the primary site for metabolising and detoxifying exogenous chemicals and drugs (Cichoż-Lach and Michalak, 2014[11]; Liu et al., 2015[40]). Additionally, it has been reported that most HMs including Cd in soft tissues are deposited mainly in the liver, the primary target for Cd exposure (Mudipalli, 2007[43]; Baba et al., 2013[6]).

Liver injury is a serious effect of exposure to Cd and positive relationship between fatty liver disease and soil Cd levels has been reported in individuals exposed to Cd (Lin et al., 2017[38]). Exposure to Cd is linked to type 2 diabetes and other metabolic alterations (Hildebrand et al., 2019[20]), and liver steatosis, fibrosis, cirrhosis and cancer were associated with high blood Cd levels (Kazi et al., 2012[29]; Chung et al., 2020[10]). Hepatotoxicity induced by Cd is closely associated with OS-mediated DNA damage and apoptosis (Skipper et al., 2016[60]). Cd-mediated ROS generation and OS disrupt cellular redox homeostasis and activate inflammatory responses (Liu et al., 2009[39]). Excess ROS can activate several signaling molecules such as nuclear factor-kappaB (NF-κB) and NLRP3 (NOD-, LRR- and pyrin domain-containing protein 3) followed by the release of inflammatory mediators and cell death (Kelley et al., 2019[30]). NF-κB-mediated NLRP3 activation promotes caspase-1 that cleaves pro-interleukin (IL)-1β into the mature form. Consequent to IL-1β release, genes involved in several disease processes are upregulated and the resultant endothelial cell response facilitates immune cells infiltration to the damaged tissue (Dinarello, 2009[15]). Therefore, attenuation of OS and the inflammatory response provoked by Cd can protect the liver against injury.

Natural compounds from plants have shown promising antioxidant, anti-inflammatory and hepatoprotective properties (Sayed et al., 2020[58]; Simental-Mendía et al., 2021[59]). The sesquiterpene alcohol farnesol (FAR) has shown promising anti-inflammatory, antioxidant, and cytoprotective efficacies (Jahangir et al., 2006[24]; Qamar and Sultana, 2008[47]; Jung et al., 2018[26]). FAR is predominantly found in essential oils of ambrette seeds, cyclamen, rose, and citronella, and present in many aromatic plants (Jung et al., 2018[26]). Several preclinical studies have demonstrated the anti-cancer, anti-inflammatory, nephroprotective, neuroprotective and antioxidant properties of FAR *in vivo* and *in vitro* (Jahangir et al., 2006[24]; Santhanasabapathy and Sudhandiran, 2015[54]; Jung et al., 2018[26]; Abukhalil et al., 2020[3]). We have reported the beneficial effects of FAR against OS and liver injury associated with hypercholesterolemia (Abukhalil et al., 2020[3]). In an acetaminophen (APAP) hepatotoxicity murine model, FAR prevented liver injury and suppressed OS and inflammation (Gayatri Devi and Ezhilarasan, 2023[19]). Despite the reported potential benefits of FAR in various conditions, its efficacy against Cd hepatotoxicity has not been explored yet. This study used a rat model of Cd hepatotoxicity to evaluate the effect of FAR on OS, inflammation, and apoptosis, with a focus on NF-κB/NRLP3 inflammasome axis and transforming growth factor-β (TGF-β)/Smad3 signaling.

## Materials and Methods

### Animals and treatments

Twenty-four 10-12 week old male Wistar rats (180-200 g), obtained from the Modern Veterinary Office for Laboratory Animals (Cairo, Egypt) were included in this investigation. The animals were kept under standard temperature (22 ± 1 °C) and humidity (50-60 %) on a 12 h dark-light cycle with *ad libitum* standard food and water. The rats were allocated into four groups (*n* = 6):

Groups I: received vehicles.

Group II: received 10 mg/kg FAR (Sigma, USA) (Abukhalil et al., 2020[3]).

Group III: received a single intraperitoneal (i.p.) injection of CdCl_2_ (1.2 mg/kg) (de Lima et al., 2020[14]) (Sigma, USA).

Group IV: received 10 mg/kg FAR and a single i.p. injection of CdCl_2_ (1.2 mg/kg) (de Lima et al., 2020[14]) (Sigma, USA).

FAR was administered by oral gavage for 14 days and CdCl_2_ was injected on day 7. 0.5 % carboxymethyl cellulose (10 ml/kg body weight) and 0.9 % saline (5 ml/kg body weight) were used as vehicles for FAR and CdCl_2_, respectively. Groups I and II received a single i.p. injection of 0.9 % saline on day 7. Blood was collected via cardiac puncture under ketamine/xylazine anesthesia at the end of the experiment. The rats were dissected, and the liver was excised. Samples were collected on 10 % neutral buffered formalin (NBF) whereas others were homogenized in cold Tris-HCl buffer (10 mM, pH = 7.4), centrifuged and the supernatant was collected and stored at -80 °C. Another set of samples was kept at -80 °C.

### Biochemical assays

Transaminases (aspartate aminotransferase (AST) and alanine aminotransferase (ALT)), alkaline phosphatase (ALP), lactate dehydrogenase (LDH), and albumin were measured in serum, and liver malondialdehyde (MDA), superoxide dismutase (SOD), GSH, nitric oxide (NO), glutathione peroxidase (GPx) and catalase (CAT) were measured using Bio-diagnostic (Egypt) kits. NF-κB p65 (Cusabio, China), IL-1β, IL-6, and tumor necrosis factor (TNF)-α (ELabscience, China) were assayed using ELISA kits.

### Histopathology and immunohistochemical investigations

Liver samples fixed in 10 % NBF for 24 h were dehydrated in ethanol, cleared in xylene, and infiltrated in pure soft paraffin followed by embedding in paraffin. 5-µm sections were cut and stained with hematoxylin & eosin (H&E), periodic acid-Schiff (PAS), and Sirius red. Other sections were dewaxed, and processed through rehydration, immersion in 0.05 M citrate buffer (pH 6.8), and treatment with 0.3 % H_2_O_2_ and protein block. The prepared sections were incubated with anti- peroxisome proliferator-activated receptor γ (PPARγ), anti-inducible NO synthase (iNOS), and anti-cleaved caspase-3 (Biospes, China) overnight at 4 °C, followed by the secondary antibody for 1 h. DAB in H_2_O_2_, followed by counterstaining with hematoxylin were employed to develop color and intensity was measured (6/rat) using ImageJ (NIH, USA).

### Western blotting

Liver samples were homogenized in RIPA buffer supplemented with phosphatase/proteinase inhibitors, centrifuged and the supernatant was separated for protein assay using Bradford reagent. Forty µg protein was subjected to SDS-PAGE followed by transfer onto PVDF membranes and blocking with 5 % BSA. The membranes were incubated with anti-NLRP3, anti-ASC, anti-caspase-1, anti-TGF-β, anti-Smad3, anti-phosphoSmad3, anti-α-smooth muscle actin (SMA), and anti-β-actin overnight at 4 °C, followed by washing and incubation with secondary antibodies for 1 h at room temperature. After washing, the bands were developed, and the band intensity was determined using ImageJ (NIH, USA).

### Molecular docking

The affinity of FAR towards ASC1 pyrin domain (PYD) domain (PDB: 1UCP), NLRP3 PYD domain (PDB: 3QF2), TGF-β (PDB ID: 6B8Y), and PPARγ (PDB ID: 2P4Y) was explored using PyRx virtual screening software (version 0.8) (Dallakyan and Olson 2015[13]). The protein targets were prepared using Autodock Tools (ADT; v1.5.6). PyMOL (v2.3.2) was used for molecular visualization and binding mode inspection, and LigPlot (v2.2.8) (Wallace et al., 1995[67]) was used to obtain protein-ligand interactions.

### Statistical analysis

The data of the study are expressed as mean ± standard deviation (SD). Statistical analysis and group comparisons were carried out using one-way ANOVA and Tukey's tests. A P value <0.05 was considered significant.

## Results

### FAR prevents Cd-induced liver injury

**Figure 1:**
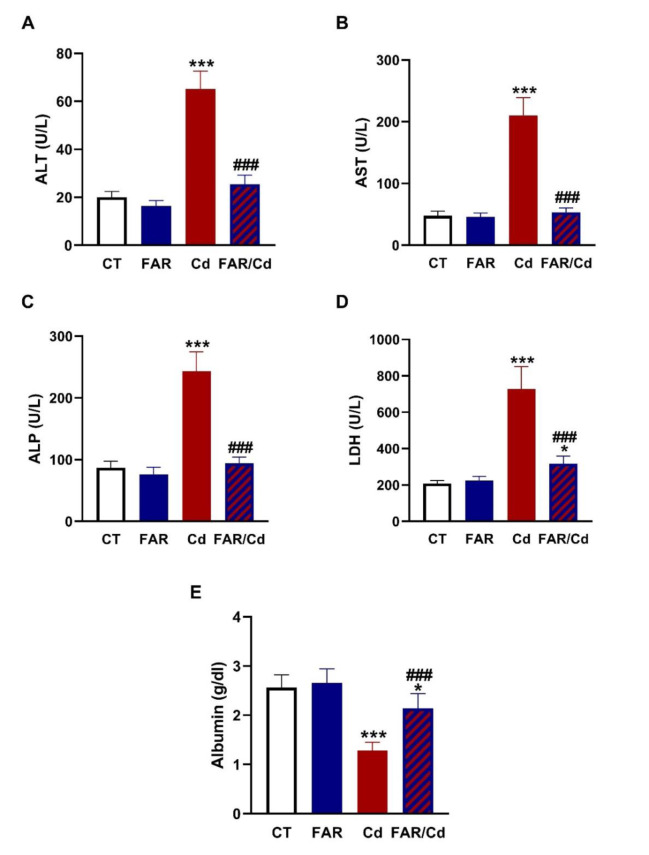
FAR ameliorated serum ALT (A), AST (B), ALP (C), LDH (D), and albumin (E) in Cd-administered rats. Data are mean ± SD, (*n* = 6). *P<0.05 and ***P<0.001 versus Control. ^###^P<0.001 versus Cd.

**Figure 2:**
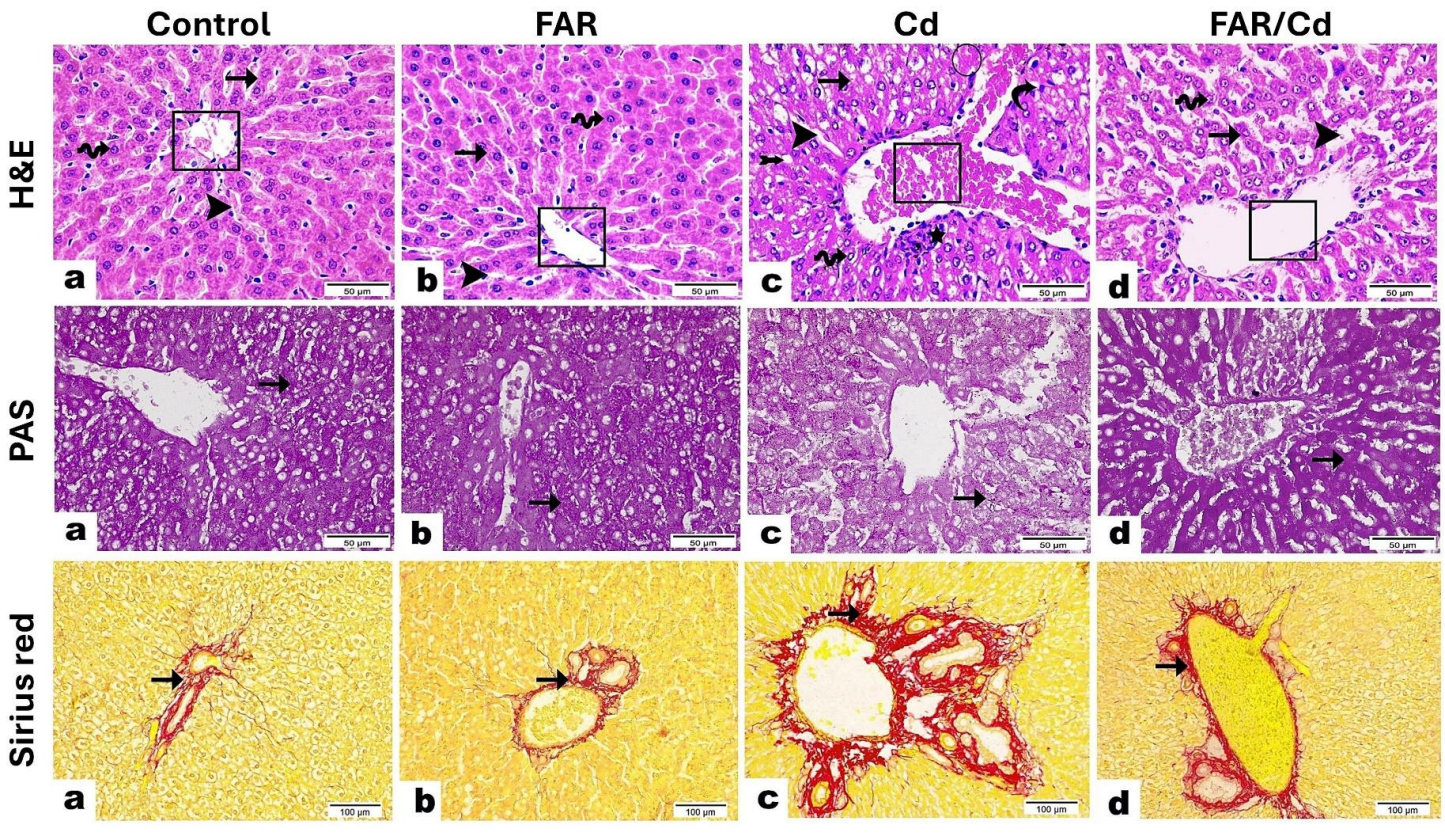
Photomicrographs showing the hepatoprotective effect of FAR on histopathological alterations induced by Cd. H&E: Liver sections from control (a) and FAR-treated (b) rats showing normal central vein (cube) and hepatocytes with central vesicular nucleus (wave arrow) arranged in regular cords (arrow) and separated by blood sinusoids (arrowhead); Cd-administered group (c) showing necrotic area (circle), loss of hepatic cord uniformity (arrow), central vein congestion (cube) and dilated sinusoids (arrowhead), hydropic degeneration with cytoplasmic vacuolation (wave arrow), deep basophilic pyknotic nuclei of hepatocytes (curvy arrow), inflammatory cell infiltration (star), and accumulated fat cells (arrow with tail); and Cd-administered rats treated with FAR (d) showing intact central vein (cube), less congestion (arrowhead), and regular hepatic cords (arrow), and hepatocytes with acidophilic cytoplasm and central nuclei (wave arrow). (x400, Scale bar= 50 µm). PAS: Liver sections from control (a) and FAR-treated (b) rats showing marked intense positive PAS staining of most hepatocytes (arrow); Cd-administered group (c) showing declined intensity of PAS staining (arrow); and Cd-administered rats treated with FAR (d) showing marked increase in staining intensity (arrow). (x400, Scale bar= 50 µm). Sirius red: Liver sections from control (a) and FAR-treated (b) rats showing little collagen encircling the portal area (arrow); Cd-administered group (c) showing high amount of collagen (arrow); and Cd-administered rats treated with FAR (d) showing noticeable decline in collagen fibers (arrow). (x200, Scale bar= 100 µm).

The biochemical findings revealed remarkable elevation in circulating ALT, AST, ALP, and LDH activities in Cd-administered rats (P<0.001) as depicted in Figure 1A-D(Fig. 1). Serum albumin was conversely decreased in rats challenged with Cd (Figure 1E(Fig. 1)). FAR effectively ameliorated the assayed circulating enzymes and albumin in Cd-administered rats. Examination of stained sections supported the protective effect of FAR on Cd-provoked liver injury (Figure 2(Fig. 2)). H&E, PAS and Sirius red staining showed normal structure of hepatocytes and sinusoids, and normal amount of collagen fibers in control (Figure 2a(Fig. 2)) and FAR-administered rats (Figure 2b(Fig. 2)). Cd induced severe alterations, including damage of hepatic cord uniformity, noticeable necrotic changes, inflammatory cell infiltration, dilated blood sinusoids, hydropic degeneration with cytoplasmic vacuolation, deep basophilic pyknotic nuclei of hepatocytes, observable accumulated fat cells, and excessive collagen deposition (Figure 2c(Fig. 2)). Cd-challenged rats treated with FAR exhibited noticeable recovery represented by regular hepatic cords, integral central vein, a lesser extent of congested sinusoids, mostly normal hepatocytes, and declined in collagen deposition (Figure 2d(Fig. 2)).

### FAR attenuates Cd-induced liver oxidative stress

**Figure 3:**
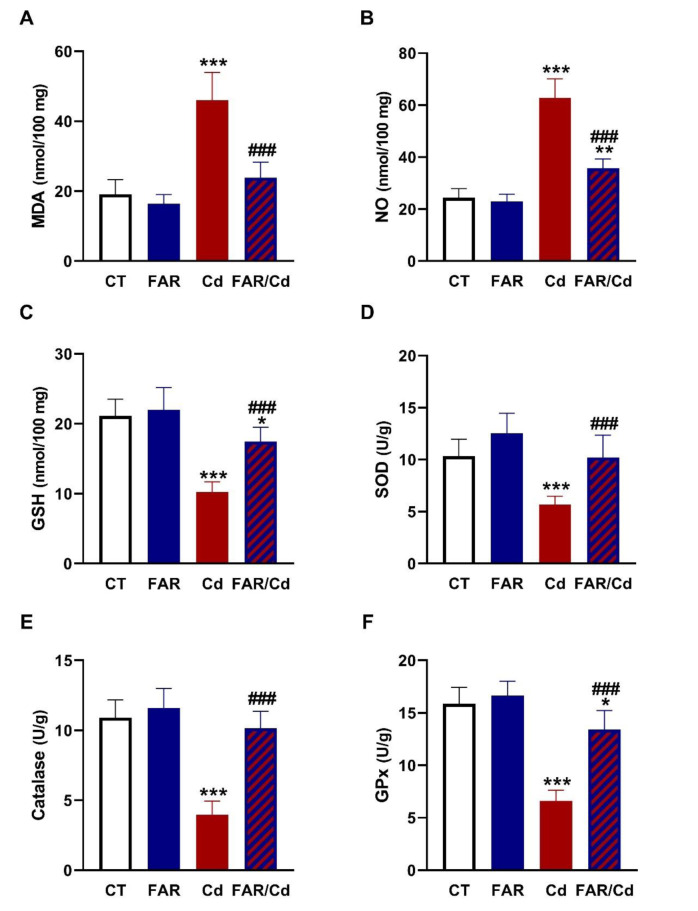
FAR ameliorated MDA (A), NO (B), GSH (C), SOD (D), catalase (E), and GPx (F) in liver of Cd-administered rats. Data are mean ± SD, (*n* = 6). *P<0.05, **P<0.01, and ***P<0.001 versus Control. ^###^P<0.001 versus Cd.

Cd administration resulted in increased hepatic MDA (Figure 3A(Fig. 3)) and NO (Figure 3B(Fig. 3)), and decreased GSH (Figure 3C(Fig. 3)), SOD (Figure 3D(Fig. 3)), CAT (Figure 3E(Fig. 3)), and GPx (Figure 3F(Fig. 3)) remarkably (P<0.001) as compared to the control group. Although exerted no effect in normal rats, FAR decreased MDA and NO, and enhanced GSH, SOD, CAT and GPx in Cd-challenged rats.

### FAR suppresses NF-κB/NLRP3 inflammasome axis and attenuates Cd-induced liver inflammation and apoptosis

**Figure 4:**
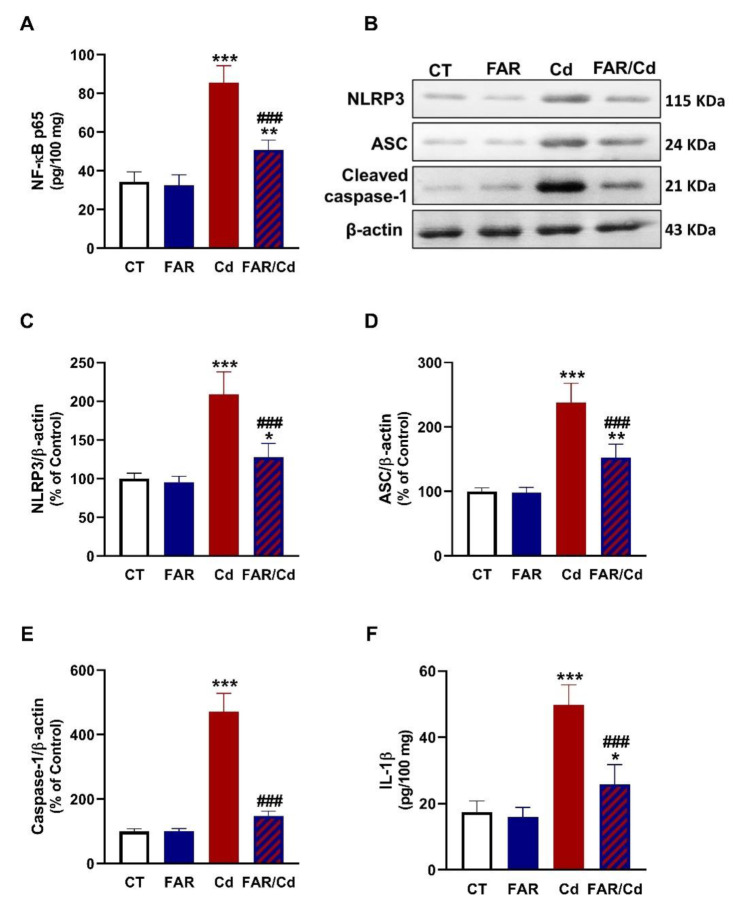
FAR downregulated NF-κB/NLRP3 inflammasome axis in liver of Cd-administered rats. FAR decreased liver NF-κB p65 (A), NLRP3, ASC, cleaved caspase-1, and IL-1β (B-F). Data are mean ± SD, (*n* = 6). *P<0.05, **P<0.01 and ***P<0.001 versus Control. ^###^P<0.001 versus Cd.

**Figure 5:**
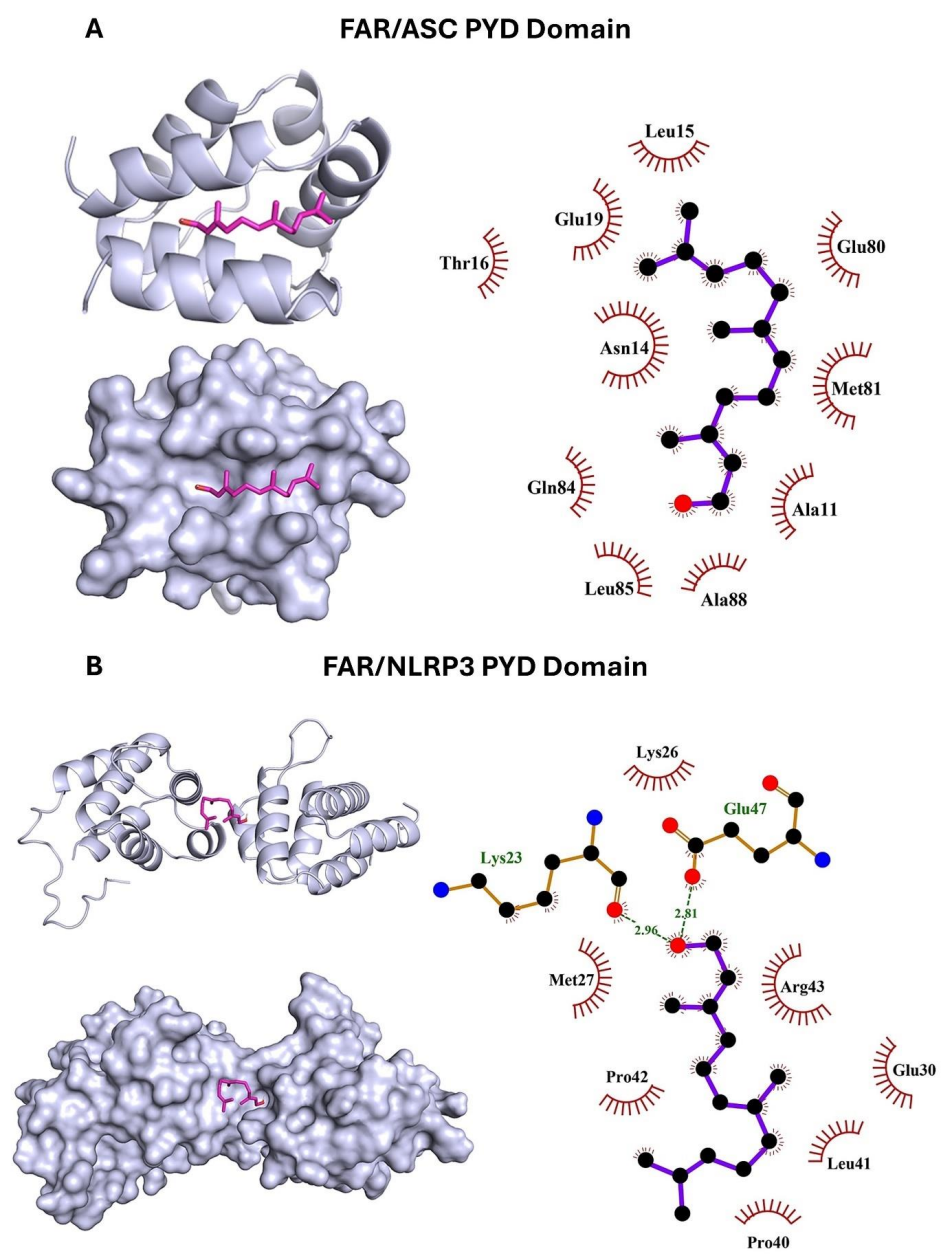
Molecular docking of FAR with ASC PYD (A) and NLRP3 PYD (B) domains showing the crystal structure and amino acid residues involved in polar bonding and hydrophobic interactions.

**Figure 6:**
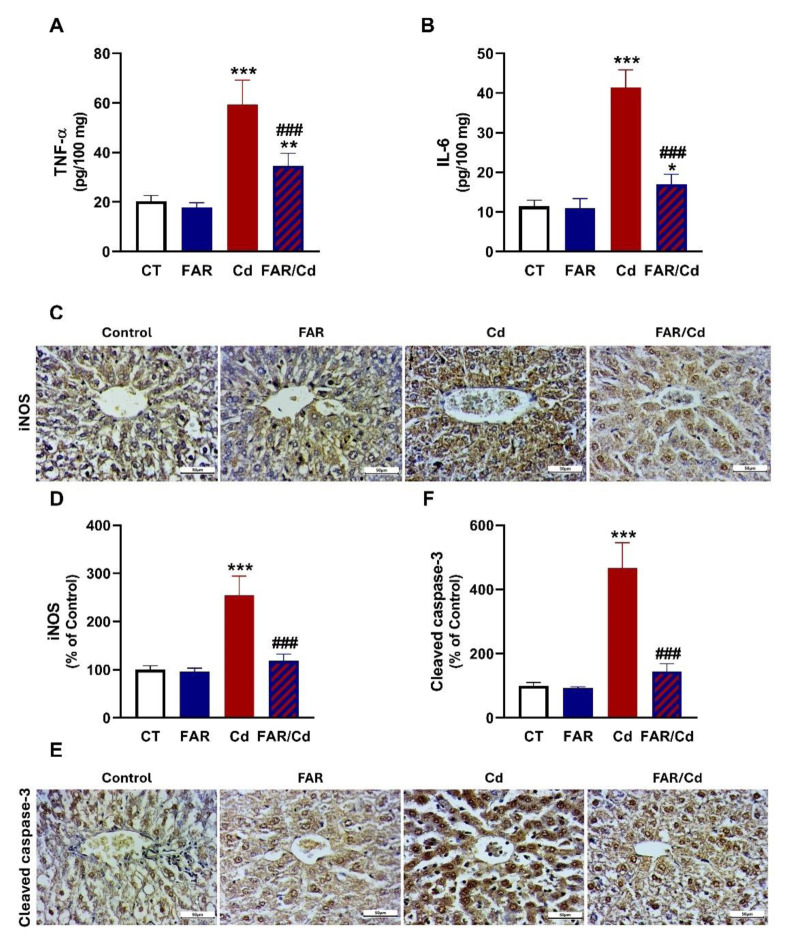
FAR ameliorated liver TNF-α (A), IL-6 (B), iNOS (C-D), and cleaved caspase-3 (E-F) in Cd-administered rats. Data are mean ± SD, (*n* = 6). *P<0.05, **P<0.01 and ***P<0.001 versus Control. ^###^P<0.001 versus Cd.

The effect of Cd and/or FAR on NF-κB/NLRP3 inflammasome axis was evaluated using immunoassays (Figure 4(Fig. 4)), and molecular docking was employed to determine the binding affinity of FAR with NLRP3 and ASC PYD domains (Figure 5(Fig. 5)). NF-κB p65, NLRP3, ASC, caspase-1, and IL-1β (Figure 4A-F(Fig. 4)) were upregulated in Cd-administered rat liver (P<0.001). FAR suppressed NF-κB p65, NLRP3, ASC, cleaved caspase-1, and IL-1β in Cd-intoxicated rats. *In silico* examination showed the binding of FAR with 10 amino acid residues in ASC PYD domain via hydrophobic interactions (Figure 5A(Fig. 5) & Table 1(Tab. 1)). The interaction between FAR and NLRP3 PYD domain included two polar bonds and 7 hydrophobic interactions (Figure 5B(Fig. 5) & Table 1(Tab. 1)). As shown in Figure 6(Fig. 6), FAR ameliorated inflammatory and apoptosis mediators in Cd-administered rat liver. Cd increased liver TNF-α (Figure 6A(Fig. 6)), IL-6 (Figure 6B(Fig. 6)), iNOS (Figure 6C-D(Fig. 6)), and cleaved caspase-3 (Figure 6E-F(Fig. 6)). Treatment with FAR significantly downregulated all assayed pro-inflammatory and apoptosis markers in Cd-administered rats.

### FAR downregulates TGF-β/Smad3 signaling in liver of Cd-administered rats

**Figure 7:**
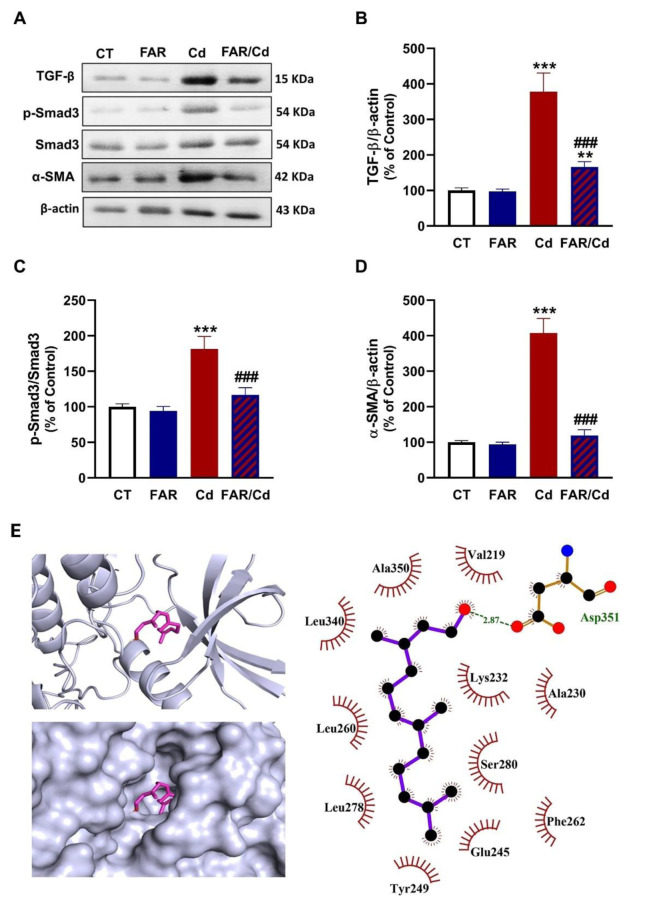
FAR downregulated TGF-β/Smad3 signaling in Cd-administered rats. FAR decreased liver TGF-β (A, B), Smad3 phosphorylation (A, C), and α-SMA (A, D). Data are mean ± SD, (*n* = 6). **P<0.01 and ***P<0.001 versus Control. ^###^P<0.001 versus Cd. (E) Molecular docking of FAR with TGF-β.

Cd upregulated liver TGF-β, Smad3 phosphorylation and α-SMA in the liver of Cd-administered rats (P<0.001; Figure 7A-D(Fig. 7)). FAR downregulated TGF-β and α-SMA expression and Smad3 phosphorylation in Cd-administered rats (P<0.001). The affinity of FAR towards TGF-β depicted in Figure 7E(Fig. 7) and Table 1(Tab. 1) demonstrated the involvement of 11 amino acid residues in hydrophobic interaction in addition to one polar bond.

### FAR upregulates liver PPARγ in Cd-administered rats

**Figure 8:**
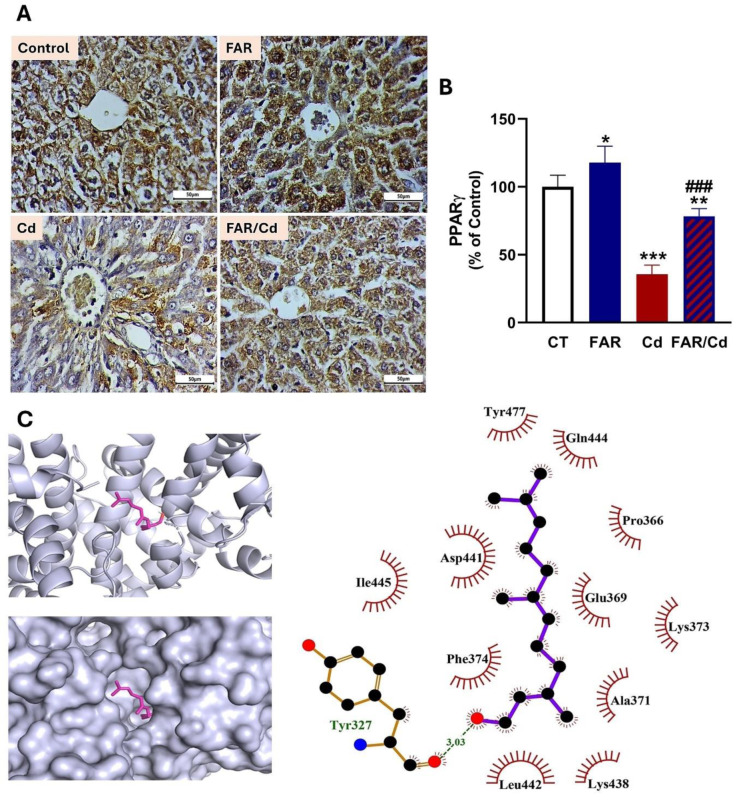
FAR increased PPARγ in the liver of normal and Cd-administered rats (A-B). Data are mean ± SD, (*n* = 6). *P<0.05, **P<0.01 and ***P<0.001 versus Control. ^###^P<0.001 versus Cd. (C) Molecular docking of FAR with PPARγ.

Liver PPARγ showed remarkable downregulation in Cd-administered rats (P<0.001) as depicted in Figure 8A-B(Fig. 8). FAR significantly upregulated liver PPARγ in rats challenged with Cd (P<0.001). *In silico* data showed hydrophobic interactions and one polar bond in the complex of FAR and PPARγ (Figure 8C(Fig. 8) & Table 1(Tab. 1)).

See also the supplementary data.

## Discussion

Exposure to HMs and other chemical agents that poses toxic effects is a major concern worldwide. Cd enters the body through different routes and humans and animals could be exposed to this HM via multiple sources (Järup and Åkesson, 2009[25]; Rzigalinski and Strobl, 2009[53]; Friberg et al., 2019[18]). Liver injury is a serious hazard of Cd exposure and liver disorders, including steatosis, fibrosis, cirrhosis, and cancer were associated with high blood Cd levels (Kazi et al., 2012[29]; Chung et al., 2020[10]; Renu et al., 2021[51]). Inflammation and OS are central in Cd toxicity and mitigation of these processes could be effective in protecting the liver and other organs against injury. Here, we demonstrated the efficacy of FAR to attenuate OS, inflammation and liver injury provoked by Cd in rats.

Cd administration resulted in elevated serum AST, ALT, ALP, and LDH activities and remarkable reduction in albumin, demonstrating hepatocyte injury. These findings are in agreement with other recent investigations demonstrating elevated serum transaminases following exposure to Cd (Renugadevi and Prabu, 2010[52]; Abu-El-Zahab et al., 2019[2]). Hepatocyte dysfunction and injury are associated with elevated serum hepatic biomarkers resulting from hepatocyte membrane damage and leakage of the contents. Elevated levels of AST, ALT, LDH, and ALP are obvious parameters to detect hepatic injury. Similar to animal studies (Renugadevi and Prabu, 2010[52]; Abu-El-Zahab et al., 2019[2]), exposure to Cd resulted in elevated circulating transaminases in humans (Kang et al., 2013[27]), a finding that was closely associated with Cd levels (Hyder et al., 2013[21]). Cd decreased serum albumin and this could be associated with Cd-induced decline in hepatic Klotho-methylation (Yu et al., 2020[71]). The determined biochemical parameters were in line with the histopathological examination which revealed severe alterations of liver structures, including remarkable necrosis, congestion, vacuolization, severe hydropic degeneration with obvious inflammatory cell infiltration and fatty degenerative changes, and high amounts of collagen deposition in Cd-administered group. Cd hepatotoxicity is associated with several structural alterations, including inflammatory cells infiltration, hyperplasia, necrosis, and apoptosis (Tzirogiannis et al., 2004[63]; Milton Prabu et al., 2012[41]). FAR supplementation prevented Cd-induced hepatocyte injury manifested by ameliorated serum transaminases and albumin, and improved tissue histological architecture. These findings support previous reports showing the hepatoprotective activity of FAR (Abukhalil et al., 2020[3]; Gayatri Devi and Ezhilarasan, 2023[19]). FAR decreased serum transaminases, LDH, and ALP, and ameliorated histopathological alterations, in particular, the fat deposition induced by hypercholesterolemia in rats (Abukhalil et al., 2020[3]). In mice challenged with APAP, FAR decreased serum ALT and AST and prevented hepatocyte injury as revealed by histopathological examination (Gayatri Devi and Ezhilarasan, 2023[19]). These findings along with our study demonstrated the efficacy of FAR to protect against hepatotoxic drugs and chemicals.

Given the role of OS and inflammation in Cd toxicity (Lin et al., 2007[37]; Liu et al., 2009[39]) and the reported antioxidant and anti-inflammatory efficacies of FAR (Jahangir et al., 2006[24]; Qamar and Sultana, 2008[47]; Jung et al., 2018[26]), the hepatoprotective role of FAR in this study could be directly ascribed to attenuation of OS and inflammation. Here, Cd caused OS in the liver as shown by elevated MDA and NO and declined GSH and antioxidant enzymes. These findings are consistent with our previous and other investigators findings of Cd-induced OS in different tissues (Sarkar et al., 1995[56]; Beytut and Aksakal, 2002[7]; Liu et al., 2015[40]; Alruhaimi et al., 2023[4]). Moreover, it has been demonstrated that exposure to Cd is associated with ROS generation, increased LPO levels, and antioxidants reduction (Liu et al., 2009[39]). The ionic form of Cd enters hepatocytes via binding to Fe^2+ ^and Zn^2+^ transporters or through voltage-gated Ca^2+^ channels. Additionally, it forms complexes with MT and the complexes enter hepatocytes via receptor-mediated endocytosis (Wolff et al., 2006[68]; Abouhamed et al., 2007[1]). Within hepatocytes, Cd is liberated from its complexes with MT through the action of lysosomes (Wolff et al., 2006[68]; Abouhamed et al., 2007[1]). Cd doesn't generate ROS via redox reactions, but produces H_2_O_2_, • O_2_, and • OH indirectly via Fenton-type reactions and other reactions provoked via Cd-mediated liberation of unbound iron (Casalino et al., 1997[9]; Ikediobi et al., 2004[23]; Cuypers et al., 2010[12]). The generated ROS can damage cellular macromolecules, resulting in cell death, and • O_2_ can react with NO to produce peroxynitrite that oxidizes DNA and increases ROS (Pacher et al., 2007[46]). Besides ROS generation, Cd causes OS through direct binding to the sulfhydryl groups of GSH and other proteins, leading to depletion of antioxidant defenses (Renu et al., 2021[51]). Furthermore, mitochondrial dysfunction mediated via Cd accumulation further increases ROS generation (Qi et al., 2020[48]).

OS is associated with inflammation and their role in Cd hepatotoxicity was acknowledged (Liu et al., 2015[40]). Cytokines and inflammatory mediators are essential tissue markers of inflammatory responses inflicted by environmental agents, including Cd (Liu et al., 2015[40]). Excess ROS provoked by Cd can activate several molecules, including NF-κB which acts as a master regulator of inflammation and immune homeostasis in different tissues by controlling large number of important pro-inflammatory mediators (Mitchell and Carmody, 2018[42]). Accordingly, Cd upregulated hepatic NF-κB p65, IL-6, TNF-α, and iNOS, demonstrating an inflammatory response. Our findings are consistent with previous findings on Cd-induced inflammatory responses in hepatocytes (Liu et al., 2015[40]) and human hepatoma cell line HepG2 (Souza et al., 2004[61]). The produced cytokines in conjunction with ROS promote apoptotic cell death mediated via mitochondrial damage. In this context, Cd-induced apoptosis in human hepatocytes was associated with mitochondrial dysfunction and damage (Lasfer et al., 2008[34]). The study of Souza et al. (2004[61]) showed the efficacy of anti-TNF-α antibodies and N-acetylcysteine to downregulate NF-κB p65, IL-1β, and IL-6 and prevent apoptosis in Cd-challenged hepatocytes *in vitro*. Excessive ROS and cytokines can damage cellular organelles, including mitochondria, resulting in outflow of cytochrome *c* and subsequent activation of pro-apoptotic proteins and initiation of apoptosis (Redza-Dutordoir and Averill-Bates, 2016[49]). Activated caspase-3 is a marker of cellular apoptosis as it acts primarily as the executioner to initiate the apoptotic process (Redza-Dutordoir and Averill-Bates, 2016[49]). In accordance, cleaved caspase-3 was elevated in the liver of Cd-administered rats in this study. Moreover, Cd exposure upregulated NLRP3, ASC, IL-1β, and caspase-1 which along with the upregulated NF-κB pinpointing activation of this inflammatory axis. ROS-provoked NF-κB is strongly correlated with NLRP3 inflammasome activation in many diseases (Kelley et al., 2019[30]). NLRP3 could also be activated by ROS to initiate an inflammatory response manifested by elevated IL-1β (Kelley et al., 2019[30]). Consequently, genes implicated in multiple disease processes are upregulated and the resultant endothelial cell response facilitates immune cells infiltration to the damaged tissue (Dinarello, 2009[15]). NLRP3 consists of a central nucleotide-binding and oligomerization domain that possesses ATPase activity necessary for oligomerization (Duncan et al., 2007[16]), C-terminal leucine-rich repeat (LRR) domain, and PYD. PYD of NLRP3 initiates the inflammasome assembly by interacting with PYD of ASC (Vajjhala et al., 2012[64]). Caspase-1 is then recruited and activated leading to cleavage of pro-IL-1β to IL-1β, and gasdermin D resulting in the formation of pores in the plasma membrane followed by cell death (Kelley et al., 2019[30]). In line with our study, Cao et al. (2022[8]) demonstrated upregulation of NLRP3 and IL-1β, and inflammation and cell death in the liver of ducks supplemented with Cd. Very recently, we revealed activation of NLRP3 in the heart of Cd-exposed mice (Antar et al., 2024[5]).

Owing to the role of OS and inflammation in mediating Cd hepatotoxicity and cell death, the hepatoprotective efficacy of FAR is therefore directly linked to suppression of OS and inflammatory response. FAR decreased MDA, NO, pro-inflammatory mediators, and caspase-3 and suppressed NF-κB/NLRP3 inflammasome axis in Cd-administered rats. The efficacy of FAR against liver injury was accompanied with decreased LPO, IL-1β, IL-6, and TNF-α, and enhanced antioxidants in experimental hypercholesterolemia (Abukhalil et al., 2020[3]) and APAP hepatotoxicity (Gayatri Devi and Ezhilarasan, 2023[19]). Other studies have supported the antioxidant and anti-inflammatory role of FAR. For instance, it downregulated TNF-α and suppressed inflammation in asthmatic mice lung (Ku and Lin, 2015[33]), and ameliorated MDA and CAT activity and prevented inflammation in a rat model of cigarette smoke extract-induced lung injury (Qamar and Sultana, 2008[47]). FAR reduced gliosis-associated pro-inflammatory mediators in mice (Santhanasabapathy et al., 2015[55]), and attenuated OS and inflammation in experimental nephrotoxicity (Jahangir et al., 2006[24]) and primary human renal epithelial cells (Müller et al., 2023[44]), respectively. It has also shown potent effect on caspase-3 in rats with colonic damage (Khan and Sultana, 2011[32]). These findings along with the current study demonstrated the potent antioxidant, anti-inflammatory, and cytoprotective efficacies of FAR in the liver of rats challenged with Cd. In addition, this study introduced novel information that suppression of the NF-κB/NLRP3 inflammasome axis is involved in the protective mechanism of FAR against Cd hepatotoxicity. To gain an insight into the modulatory effect of FAR on NLRP3 inflammasome, we employed molecular docking. Our investigation revealed the affinity of FAR to bind the PYD domain of NLRP3 and ASC. FAR showed hydrophobic interactions with ASC and NLRP3 PYD domains and polar bonding with NLRP3 PYD domain suggesting its ability to inhibit the PYD-PYD interaction necessary for the inflammasome assembly and subsequent activation of caspase-1 and cell death. Our study pinpointed the ability of FAR to downregulate TGF-β/ Smad3 signaling in the liver of Cd-administered rats, thereby inhibiting collagen deposition and fibrogenesis. TGF-β is a pleiotropic cytokine with key roles in inflammation and liver fibrosis (Li and Flavell, 2008[35]). TGF-β1 release by necrotic hepatocytes activates hepatic stellate cells (HSCs) and promotes its transdifferentiation into myofibroblasts and the release of excessive amounts of collagen, while preventing its apoptosis and inhibits the degradation of extracellular matrix (ECM) (Kanzler et al., 1999[28]). Smad proteins mediate fibrogenesis induced by TGF-β signaling. Upon binding of TGF-β1 to its receptor, Smad2 and Smad3 are phosphorylated and form a complex with Smad4. The complex translocates into the nucleus and induces the transcription of several genes involved in fibrogenesis (Xie et al., 2014[69]). TGF-β1 can also activate NF-κB and Smads act as signal integrators that interact with NF-κB signaling (Xie et al., 2014[69]). Our study revealed upregulation of TGF-β1 and Smad3 phosphorylation along with upregulated α-SMA indicating activation and transdifferentiation of HSCs and fibrogenesis as shown in the stained sections. Circulating Cd levels are positively correlated with fibrogenesis in human subjects and in patients with liver cirrhosis (Kazi et al., 2012[29]; Chung et al., 2020[10]). By using Raman confocal imaging, Li et al. (2020[36]) demonstrated a higher collagen peak in liver samples exposed to Cd. These studies along with activated TGF-β1/Smad3 signaling explained the increase in collagen deposition in the liver of Cd-administered rats. FAR suppressed TGF-β1, α-SMA, Smad3 phosphorylation and collagen deposition, adding further explanation to its hepatoprotective mechanism. *In silico* data showed the binding affinity of FAR towards TGF-β, a finding that supports the suppressive effect of FAR on TGF-β signaling.

Given the key role of PPARγ activation in mitigating OS, inflammation and fibrogenesis, we investigated its possible involvement in the hepatoprotective efficacy of FAR. Cd-administered rats exhibited a decline in liver PPARγ, an effect that FAR prevented. Upregulation of PPARγ enhances antioxidant enzymes, inhibits ROS generation, and suppresses NF-κB by controlling its transcriptional activity, reducing p65 nuclear translocation and inhibiting IκBα degradation (Kersten et al., 2000[31]; Remels et al., 2009[50]). The effect of FAR on PPARγ has been reported in very few reports. The study of Torabi and Mo (2016[62]) showed upregulated PPARγ in T3-F442A pre-adipocyte treated with FAR. Upregulation of PPARγ was involved in the effect of FAR on the maturation of human dendritic cells (Vivas et al., 2019[66]). Here, we introduced information on the positive effect of FAR on PPARγ in rats, an effect that was supported by the *in silico* data showing the polar and hydrophobic interactions between FAR and PPARγ.

## Conclusions

This study introduced novel information on the protective efficacy of FAR on Cd hepatotoxicity. Inflammation, OS, upregulated NF-κB/NLRP3 inflammasome axis and TGF-β/Smad3 signaling and declined PPARγ were demonstrated following Cd administration. FAR attenuated OS, inflammation and liver injury, suppressed NF-κB/NLRP3 inflammasome axis and TGF-β/Smad3 signaling, and boosted PPARγ and antioxidants. FAR showed binding affinity towards NLRP3 and ASC PYD domains, TGF-β and PPARγ. Therefore, FAR effectively protected rats against Cd hepatotoxicity, and further investigations to explore other underlying mechanism(s) are recommended.

## Declaration

### Ethical approval

The animal study protocol was approved by the ethics committee of Al-Azhar University (Assiut, Egypt) (AZ-AS/PH-REC/39/24).

### Declaration of competing interest

No conflict of interest is to be declared.

### Data availability

The manuscript contains all data supporting the reported results.

### Acknowledgment

Princess Nourah bint Abdulrahman University Researchers Supporting Project Number (PNURSP2024R381), Princess Nourah bint Abdulrahman University, Riyadh, Saudi Arabia.

### Author contribution

Conceptualization, EHMH, and AMM; methodology, RSA, SMA, MAA, OAMA, MKM, EHMH, IE, and AMM; formal analysis, RSA, EHMH, and AMM; investigation, RSA, OAMA, MKM, EHMH, and AMM; resources, SMA, MAA, and IE; data curation, RSA, EHMH, and AMM; writing-original draft preparation, AMM; writing-review and editing, AMM; supervision, EHMH, and AMM; project administration, RSA, and AMM; funding acquisition, RSA. All authors have read and agreed to the published version of the manuscript.

## Supplementary Material

Supplementary data

## Figures and Tables

**Table 1:**
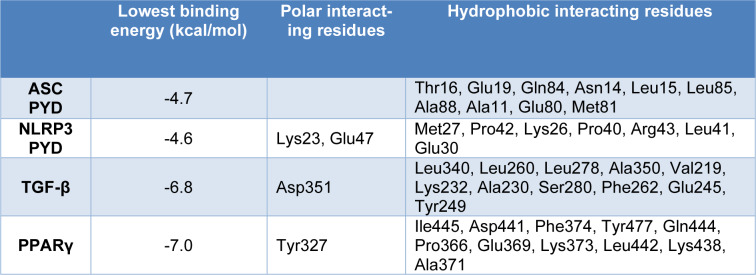
Binding affinities of FAR towards ASC and NLRP3 PYD domains, TGF-β, and PPARγ.

## References

[1] Abouhamed M, Wolff NA, Lee WK, Smith CP, Thévenod F (2007). Knockdown of endosomal/lysosomal divalent metal transporter 1 by RNA interference prevents cadmium-metallothionein-1 cytotoxicity in renal proximal tubule cells. Am J Physiol Renal Physiol.

[2] Abu-El-Zahab HSH, Hamza RZ, Montaser MM, El-Mahdi MM, Al-Harthi WA (2019). Antioxidant, antiapoptotic, antigenotoxic, and hepatic ameliorative effects of L-carnitine and selenium on cadmium-induced hepatotoxicity and alterations in liver cell structure in male mice. Ecotoxicol Environ Saf.

[3] Abukhalil MH, Hussein OE, Bin-Jumah M, Saghir SAM, Germoush MO, Elgebaly HA (2020). Farnesol attenuates oxidative stress and liver injury and modulates fatty acid synthase and acetyl-CoA carboxylase in high cholesterol-fed rats. Environ Sci Pollut Res.

[4] Alruhaimi RS, Hassanein EH, Bin-Jumah MN, Mahmoud AM (2023). Cadmium cardiotoxicity is associated with oxidative stress and upregulated TLR-4/NF-kB pathway in rats;protective role of agomelatine. Food Chem Toxicol.

[5] Antar SA, Abdo W, Helal AI, Abduh MS, Hakami ZH, Germoush MO (2024). Coenzyme Q10 mitigates cadmium cardiotoxicity by downregulating NF-kB/ NLRP3 inflammasome axis and attenuating oxidative stress in mice. Life Sci.

[6] Baba H, Tsuneyama K, Yazaki M, Nagata K, Minamisaka T, Tsuda T (2013). The liver in itai-itai disease (chronic cadmium poisoning): pathological features and metallothionein expression. Mod Pathol.

[7] Beytut E, Aksakal M (2002). The effect of long-term supplemental dietary cadmium on lipid peroxidation and the antioxidant system in the liver and kidneys of rabbits. Turk J Vet Anim Sci.

[8] Cao P, Nie G, Luo J, Hu R, Li G, Hu G (2022). Cadmium and molybdenum co-induce pyroptosis and apoptosis via the PTEN/PI3K/AKT axis in the livers of Shaoxing ducks (Anas platyrhynchos). Food Funct.

[9] Casalino E, Sblano C, Landriscina C (1997). Enzyme activity alteration by cadmium administration to rats: the possibility of iron involvement in lipid peroxidation. Arch Biochem Biophys.

[10] Chung SM, Moon JS, Yoon JS, Won KC, Lee HW (2020). The sex-specific effects of blood lead, mercury, and cadmium levels on hepatic steatosis and fibrosis: Korean nationwide cross-sectional study. J Trace Elem Med Biol.

[11] Cichoż-Lach H, Michalak A (2014). Oxidative stress as a crucial factor in liver diseases. World J Gastroenterol.

[12] Cuypers A, Plusquin M, Remans T, Jozefczak M, Keunen E, Gielen H (2010). Cadmium stress: an oxidative challenge. Biometals.

[13] Dallakyan S, Olson AJ (2015). Small-molecule library screening by docking with PyRx. Methods Mol Biol.

[14] de Lima EC, de Moura CFG, Silva MJD, Vilegas W, Santamarina AB, Pisani LP (2020). Therapeutical properties of Mimosa caesalpiniifolia in rat liver intoxicated with cadmium. Environ Sci Pollut Res Int.

[15] Dinarello CA (2009). Immunological and inflammatory functions of the interleukin-1 family. Annu Rev Immunol.

[16] Duncan JA, Bergstralh DT, Wang Y, Willingham SB, Ye Z, Zimmermann AG (2007). Cryopyrin/NALP3 binds ATP/dATP, is an ATPase, and requires ATP binding to mediate inflammatory signaling. Proc Natl Acad Sci U S A.

[17] Egger AE, Grabmann G, Gollmann-Tepeköylü C, Pechriggl EJ, Artner C, Türkcan A (2019). Chemical imaging and assessment of cadmium distribution in the human body. Metallomics.

[18] Friberg LT, Elinder C-G, Kjellstrom T, Nordberg GF (2019). Cadmium and health: A toxicological and epidemiological appraisal: Volume 2: Effects and response: Boca Raton, FL: CRC Press, 2019.

[19] Gayatri Devi R, Ezhilarasan D (2023). Concurrent administration of farnesol protects acetaminophen-induced acute hepatic necrosis in mice. J Biochem Mol Toxicol.

[20] Hildebrand J, Thakar S, Watts TL, Banfield L, Thabane L, Macri J (2019). The impact of environmental cadmium exposure on type 2 diabetes risk: a protocol for an overview of systematic reviews. Syst Rev.

[21] Hyder O, Chung M, Cosgrove D, Herman JM, Li Z, Firoozmand A (2013). Cadmium exposure and liver disease among US adults. J Gastrointest Surg.

[22] IARC (1993). Beryllium, cadmium, mercury, and exposures in the glass manufacturing industry. IARC Monogr Eval Carcinog Risks Hum.

[23] Ikediobi CO, Badisa VL, Ayuk-Takem LT, Latinwo LM, West J (2004). Response of antioxidant enzymes and redox metabolites to cadmium-induced oxidative stress in CRL-1439 normal rat liver cells. Int J Mol Med.

[24] Jahangir T, Khan TH, Prasad L, Sultana S (2006). Farnesol prevents Fe-NTA-mediated renal oxidative stress and early tumour promotion markers in rats. Hum Exp Toxicol.

[25] Järup L, Åkesson A (2009). Current status of cadmium as an environmental health problem. Toxicol Appl Pharmacol.

[26] Jung YY, Hwang ST, Sethi G, Fan L, Arfuso F, Ahn KS (2018). Potential anti-inflammatory and anti-cancer properties of farnesol. Molecules.

[27] Kang MY, Cho SH, Lim YH, Seo JC, Hong YC (2013). Effects of environmental cadmium exposure on liver function in adults. Occup Environ Med.

[28] Kanzler S, Lohse AW, Keil A, Henninger J, Dienes HP, Schirmacher P (1999). TGF-beta1 in liver fibrosis: an inducible transgenic mouse model to study liver fibrogenesis. Am J Physiol.

[29] Kazi TG, Kolachi NF, Afridi HI, Kazi NG, Sirajuddin, Naeemullah (2012). Effects of mineral supplementation on liver cirrhotic/cancer male patients. Biol Trace Elem Res.

[30] Kelley N, Jeltema D, Duan Y, He Y (2019). The NLRP3 inflammasome: an overview of mechanisms of activation and regulation. Int J Mol Sci.

[31] Kersten S, Desvergne B, Wahli W (2000). Roles of PPARs in health and disease. Nature.

[32] Khan R, Sultana S (2011). Farnesol attenuates 1, 2-dimethylhydrazine induced oxidative stress, inflammation and apoptotic responses in the colon of Wistar rats. Chem Biol Interact.

[33] Ku C-M, Lin J-Y (2015). Farnesol, a sesquiterpene alcohol in herbal plants, exerts anti-inflammatory and antiallergic effects on ovalbumin-sensitized and-challenged asthmatic mice. Evid Based Complement Alternat Med.

[34] Lasfer M, Vadrot N, Aoudjehane L, Conti F, Bringuier AF, Feldmann G (2008). Cadmium induces mitochondria-dependent apoptosis of normal human hepatocytes. Cell Biol Toxicol.

[35] Li MO, Flavell RA (2008). Contextual regulation of inflammation: a duet by transforming growth factor-beta and interleukin-10. Immunity.

[36] Li Y, Shen R, Wu H, Yu L, Wang Z, Wang D (2020). Liver changes induced by cadmium poisoning distinguished by confocal Raman imaging. Spectrochim Acta A Mol Biomol Spectrosc.

[37] Lin A-J, Zhang X-H, Chen M-M, Qing C (2007). Oxidative stress and DNA damages induced by cadmium accumulation. J Environ Sci (China).

[38] Lin YC, Lian IB, Kor CT, Chang CC, Su PY, Chang WT (2017). Association between soil heavy metals and fatty liver disease in men in Taiwan: a cross sectional study. BMJ Open.

[39] Liu J, Qu W, Kadiiska MB (2009). Role of oxidative stress in cadmium toxicity and carcinogenesis. Toxicol Appl Pharmacol.

[40] Liu L, Tao R, Huang J, He X, Qu L, Jin Y (2015). Hepatic oxidative stress and inflammatory responses with cadmium exposure in male mice. Environ Toxicol Pharmacol.

[41] Milton Prabu S, Muthumani M, Shagirtha K (2012). Protective effect of Piper betle leaf extract against cadmium-induced oxidative stress and hepatic dysfunction in rats. Saudi J Biol Sci.

[42] Mitchell JP, Carmody RJ (2018). NF-κB and the transcriptional control of inflammation. Int Rev Cell Mol Biol.

[43] Mudipalli A (2007). Lead hepatotoxicity & potential health effects. Indian J Med Res.

[44] Müller A, Lozoya M, Chen X, Weissig V, Nourbakhsh M (2023). Farnesol inhibits PI3 kinase signaling and inflammatory gene expression in primary human renal epithelial cells. Biomedicines.

[45] Oskarsson A, Widell A, Olsson I-M, Petersson Grawé K (2004). Cadmium in food chain and health effects in sensitive population groups. Biometals.

[46] Pacher P, Beckman JS, Liaudet L (2007). Nitric oxide and peroxynitrite in health and disease. Physiol Rev.

[47] Qamar W, Sultana S (2008). Farnesol ameliorates massive inflammation, oxidative stress and lung injury induced by intratracheal instillation of cigarette smoke extract in rats: an initial step in lung chemoprevention. Chem Biol Interact.

[48] Qi K, Ren L, Bai Z, Yan J, Deng X, Zhang J (2020). Detecting cadmium during ultrastructural characterization of hepatotoxicity. J Trace Elem Med Biol.

[49] Redza-Dutordoir M, Averill-Bates DA (2016). Activation of apoptosis signalling pathways by reactive oxygen species. Biochim Biophys Acta.

[50] Remels AH, Langen RC, Gosker HR, Russell AP, Spaapen F, Voncken JW (2009). PPARgamma inhibits NF-kappaB-dependent transcriptional activation in skeletal muscle. Am J Physiol Endocrinol Metab.

[51] Renu K, Chakraborty R, Myakala H, Koti R, Famurewa AC, Madhyastha H (2021). Molecular mechanism of heavy metals (Lead, Chromium, Arsenic, Mercury, Nickel and Cadmium) - induced hepatotoxicity – A review. Chemosphere.

[52] Renugadevi J, Prabu SM (2010). Cadmium-induced hepatotoxicity in rats and the protective effect of naringenin. Exp Toxicol Pathol.

[53] Rzigalinski BA, Strobl JS (2009). Cadmium-containing nanoparticles: perspectives on pharmacology and toxicology of quantum dots. Toxicol Appl Pharmacol.

[54] Santhanasabapathy R, Sudhandiran G (2015). Farnesol attenuates lipopolysaccharide-induced neurodegeneration in Swiss albino mice by regulating intrinsic apoptotic cascade. Brain Research.

[55] Santhanasabapathy R, Vasudevan S, Anupriya K, Pabitha R, Sudhandiran G (2015). Farnesol quells oxidative stress, reactive gliosis and inflammation during acrylamide-induced neurotoxicity: Behavioral and biochemical evidence. Neuroscience.

[56] Sarkar S, Yadav P, Trivedi R, Bansal AK, Bhatnagar D (1995). Cadmium-induced lipid peroxidation and the status of the antioxidant system in rat tissues. J Trace Elem Med Biol.

[57] Satarug S, Garrett SH, Sens MA, Sens DA (2010). Cadmium, environmental exposure, and health outcomes. Environ Health Perspect.

[58] Sayed AM, Hassanein EHM, Salem SH, Hussein OE, Mahmoud AM (2020). Flavonoids-mediated SIRT1 signaling activation in hepatic disorders. Life Sci.

[59] Simental-Mendía LE, Gamboa-Gómez CI, Guerrero-Romero F, Simental-Mendía M, Sánchez-García A, Rodríguez-Ramírez M (2021). Beneficial effects of plant-derived natural products on non-alcoholic fatty liver disease. Adv Exp Med Biol.

[60] Skipper A, Sims JN, Yedjou CG, Tchounwou PB (2016). Cadmium chloride induces DNA damage and apoptosis of human liver carcinoma cells via oxidative stress. Int J Environ Res Public Health.

[61] Souza V, del Carmen Escobar M, Gómez-Quiroz L, Bucio L, Hernández E, Cossio EC (2004). Acute cadmium exposure enhances AP-1 DNA binding and induces cytokines expression and heat shock protein 70 in HepG2 cells. Toxicology.

[62] Torabi S, Mo H (2016). Trans, trans-farnesol as a mevalonate-derived inducer of murine 3T3-F442A pre-adipocyte differentiation. Exp Biol Med (Maywood).

[63] Tzirogiannis KN, Panoutsopoulos GI, Demonakou MD, Papadimas GK, Kondyli VG, Kourentzi KT (2004). The hepatoprotective effect of putrescine against cadmium-induced acute liver injury. Arch Toxicol.

[64] Vajjhala PR, Mirams RE, Hill JM (2012). Multiple binding sites on the pyrin domain of ASC protein allow self-association and interaction with NLRP3 protein. J Biol Chem.

[65] Vesey DA (2010). Transport pathways for cadmium in the intestine and kidney proximal tubule: focus on the interaction with essential metals. Toxicol Lett.

[66] Vivas W, Leonhardt I, Hünniger K, Häder A, Marolda A, Kurzai O (2019). Multiple signaling pathways involved in human dendritic cell maturation are affected by the fungal quorum-sensing molecule farnesol. J Immunol.

[67] Wallace AC, Laskowski RA, Thornton JM (1995). LIGPLOT: a program to generate schematic diagrams of protein-ligand interactions. Protein Eng.

[68] Wolff NA, Abouhamed M, Verroust PJ, Thévenod F (2006). Megalin-dependent internalization of cadmium-metallothionein and cytotoxicity in cultured renal proximal tubule cells. J Pharmacol Exp Ther.

[69] Xie F, Zhang Z, van Dam H, Zhang L, Zhou F (2014). Regulation of TGF-β superfamily signaling by SMAD mono-ubiquitination. Cells.

[70] Young JL, Yan X, Xu J, Yin X, Zhang X, Arteel GE (2019). Cadmium and high-fat diet disrupt renal, cardiac and hepatic essential metals. Sci Rep.

[71] Yu D, Zhang L, Yu G, Nong C, Lei M, Tang J (2020). Association of liver and kidney functions with Klotho gene methylation in a population environment exposed to cadmium in China. Int J Environ Health Res.

