# Supplementary material for: The sesquiterpene alcohol farnesol mitigates cadmium hepatotoxicity by attenuating oxidative stress and NF-kappaB/NLRP3 inflammasome axis and upregulating PPARgamma in rats
Source: EXCLI J. 2024 Nov 12;23:1356–74. doi: 10.17179/excli2024-7488 (PMC11701298; doi:10.17179/excli2024-7488)

*EXCLI Journal 2024;23:1356-1374 – ISSN 1611-2156*
*Received: June 06, 2024, accepted: November 05, 2024, published: November 12, 2024*

**Supplementary data to:**

## Original article:

# THE SESQUITERPENE ALCOHOL FARNESOL MITIGATES CADMIUM HEPATOTOXICITY BY ATTENUATING OXIDATIVE STRESS AND NF-κB/NLRP3 INFLAMMASOME AXIS AND UPREGULATING PPARγ IN RATS

Reem S. Alruhaimi[1], Emad H.M. Hassanein[2], Sulaiman M. Alnasser[3], Mohammed A. Alzoghaibi[4], Omnia A.M. Abd El-Ghafar[5], Mostafa K. Mohammad[6], Ibrahim Elbagory[7], Ayman M. Mahmoud[8,9]*

1 Department of Biology, College of Science, Princess Nourah bint Abdulrahman University, Riyadh 11671, Saudi Arabia
2 Department of Pharmacology & Toxicology, Faculty of Pharmacy, Al-Azhar University-Assiut Branch, Assiut 71524, Egypt
3 Department of Pharmacology and Toxicology, College of Pharmacy, Qassim University, Qassim 51452, Saudi Arabia
4 Physiology Department, College of Medicine, King Saud University, Riyadh, 11461, Saudi Arabia
5 Department of Pharmacology and Toxicology, Faculty of Pharmacy, Nahda University, Beni-Suef 62764, Egypt
6 Department of Pharmacology and Toxicology, Faculty of Pharmacy, Badr University in Assiut, New Nasser City, West of Assiut, Assiut 71523, Egypt
7 Department of Pharmaceutics, Faculty of Pharmacy, Northern Border University, Rafha 76321, Saudi Arabia
8 Department of Life Sciences, Faculty of Science and Engineering, Manchester Metropolitan University, Manchester M1 5GD, UK
9 Molecular Physiology Division, Zoology Department, Faculty of Science, Beni-Suef University, Beni-Suef 62514, Egypt

* **Corresponding author:** Ayman M. Mahmoud, Department of Life Sciences, Faculty of Science and Engineering, Manchester Metropolitan University, Manchester M1 5GD, UK. E-Mail: a.mahmoud@mmu.ac.uk

https://dx.doi.org/10.17179/excli2024-7488

This is an Open Access article distributed under the terms of the Creative Commons Attribution License (http://creativecommons.org/licenses/by/4.0/).

EXCLI Journal 2024;23:1356-1374 – ISSN 1611-2156
Received: June 06, 2024, accepted: November 05, 2024, published: November 12, 2024

**Supplementary Table 1:** Raw data of the parameters in Figure 1, 3, 4, 6, 7 and 8

| | | Group I | Group II | Group III | Group IV |
|---|---|---|---|---|---|
| Figure 1 | ALT | 19 | 15 | 53 | 22 |
| | | 17 | 15 | 61 | 25 |
| | | 19 | 20 | 60 | 30 |
| | | 23 | 18 | 67 | 27 |
| | | 21 | 17 | 78 | 19 |
| | | 22 | 13 | 73 | 20 |
| | AST | 42 | 41 | 173 | 51 |
| | | 51 | 46 | 187 | 48 |
| | | 37 | 48 | 200 | 55 |
| | | 50 | 51 | 217 | 63 |
| | | 59 | 54 | 233 | 59 |
| | | 46 | 36 | 251 | 45 |
| | ALP | 71 | 67 | 197 | 90 |
| | | 84 | 66 | 251 | 85 |
| | | 91 | 94 | 237 | 81 |
| | | 79 | 81 | 222 | 98 |
| | | 94 | 82 | 284 | 102 |
| | | 101 | 67 | 268 | 107 |
| | LDH | 191 | 204 | 559 | 292 |
| | | 196 | 229 | 662 | 314 |
| | | 194 | 236 | 746 | 338 |
| | | 220 | 260 | 682 | 355 |
| | | 225 | 225 | 808 | 248 |
| | | 224 | 199 | 910 | 355 |
| | Albumin | 2.2 | 3.0 | 1.1 | 2.3 |
| | | 2.3 | 2.3 | 1.2 | 2.6 |
| | | 2.4 | 2.7 | 1.3 | 1.9 |
| | | 2.7 | 2.4 | 1.1 | 1.8 |
| | | 3.0 | 2.7 | 1.4 | 2.2 |
| | | 2.8 | 2.9 | 1.6 | 2.1 |
| Figure 3 | MDA | 16.1 | 19.8 | 50.4 | 18.3 |
| | | 22.4 | 18.1 | 35.6 | 20.0 |
| | | 14.8 | 16.5 | 42.6 | 29.3 |
| | | 18.5 | 17.2 | 57.9 | 28.8 |
| | | 25.8 | 12.6 | 40.9 | 22.4 |
| | | 17.1 | 14.1 | 48.9 | 24.1 |
| | NO | 21.2 | 25.6 | 52.0 | 33.0 |
| | | 25.8 | 20.4 | 69.4 | 39.4 |
| | | 27.1 | 27.0 | 61.0 | 41.0 |
| | | 29.0 | 19.0 | 72.0 | 31.6 |
| | | 22.9 | 22.5 | 64.6 | 35.2 |
| | | 20.0 | 23.2 | 58.0 | 34.5 |
| | GSH | 19.9 | 18.7 | 8.7 | 17.1 |
| | | 18.3 | 18.0 | 9.5 | 20.6 |
| | | 22.5 | 23.3 | 10.6 | 18.0 |
| | | 23.0 | 24.3 | 11.1 | 16.6 |
| | | 18.9 | 21.4 | 9.1 | 14.3 |
| | | 24.1 | 26.1 | 12.5 | 18.0 |
| | SOD | 12.0 | 14.4 | 4.7 | 10.1 |

EXCLI Journal 2024;23:1356-1374 – ISSN 1611-2156
Received: June 06, 2024, accepted: November 05, 2024, published: November 12, 2024

| | | | | | |
|---|---|---|---|---|---|
| | | 8.7 | 15.1 | 5.4 | 12.3 |
| | | 9.5 | 10.3 | 6.4 | 13.3 |
| | | 12.7 | 10.8 | 5.0 | 9.3 |
| | | 9.2 | 12.6 | 5.7 | 8.2 |
| | | 10.0 | 12.1 | 6.8 | 8.1 |
| | **CAT** | 9.4 | 10.6 | 2.6 | 9.1 |
| | | 10.9 | 11.6 | 3.8 | 10.2 |
| | | 11.4 | 9.8 | 4.5 | 8.6 |
| | | 11.1 | 12.4 | 3.1 | 10.7 |
| | | 12.9 | 13.7 | 4.5 | 12.1 |
| | | 9.7 | 11.5 | 5.3 | 10.2 |
| | **GPX** | 13.5 | 17.1 | 6.6 | 12.5 |
| | | 15.6 | 14.9 | 7.1 | 14.9 |
| | | 16.2 | 15.8 | 5.2 | 11.1 |
| | | 14.9 | 18.8 | 7.2 | 13.8 |
| | | 17.2 | 16.4 | 7.8 | 16.0 |
| | | 17.8 | 17.0 | 5.7 | 12.2 |
| **Figure 4** | **NF-kB p65** | 31.80 | 28.00 | 74.00 | 51.00 |
| | | 37.00 | 27.15 | 79.90 | 45.00 |
| | | 28.00 | 36.00 | 86.00 | 45.60 |
| | | 32.20 | 41.40 | 98.90 | 53.25 |
| | | 35.65 | 32.20 | 85.10 | 58.65 |
| | | 42.55 | 31.05 | 90.85 | 51.75 |
| | **NLRP3** | 114 | 96 | 215 | 141 |
| | | 107 | 111 | 245 | 127 |
| | | 86 | 102 | 190 | 121 |
| | | 105 | 89 | 165 | 105 |
| | | 91 | 90 | 231 | 155 |
| | | 97 | 84 | 210 | 118 |
| | **ASC** | 104 | 105 | 236 | 184 |
| | | 91 | 114 | 219 | 141 |
| | | 108 | 93 | 220 | 136 |
| | | 95 | 89 | 205 | 129 |
| | | 117 | 90 | 274 | 169 |
| | | 85 | 96 | 275 | 155 |
| | **Caspase-1** | 87 | 105 | 471 | 121 |
| | | 102 | 112 | 512 | 148 |
| | | 121 | 116 | 425 | 151 |
| | | 111 | 91 | 385 | 136 |
| | | 84 | 88 | 554 | 174 |
| | | 95 | 95 | 520 | 155 |
| | **IL-1B** | 14.9 | 18.6 | 43.3 | 35.7 |
| | | 22.7 | 20.0 | 59.8 | 32.8 |
| | | 15.8 | 13.3 | 52.2 | 24.9 |
| | | 16.7 | 12.8 | 51.1 | 20.3 |
| | | 20.3 | 15.4 | 47.9 | 18.4 |
| | | 14.1 | 16.1 | 44.2 | 22.9 |
| **Figure 6** | **TNF-a** | 20.5 | 16.8 | 74.7 | 33.2 |
| | | 18.6 | 22.7 | 53.3 | 33.6 |
| | | 25.7 | 19.2 | 55.9 | 43.7 |
| | | 21.8 | 17.4 | 63.9 | 30.2 |

EXCLI Journal 2024;23:1356-1374 – ISSN 1611-2156
Received: June 06, 2024, accepted: November 05, 2024, published: November 12, 2024

| | | | | | |
|---|---|---|---|---|---|
| | | 20.3 | 14.5 | 46.5 | 37.9 |
| | | 16.5 | 16.5 | 62.3 | 29.7 |
| | **IL-6** | 11.0 | 8.1 | 40.1 | 15.4 |
| | | 12.2 | 12.8 | 47.3 | 22.1 |
| | | 9.7 | 13.5 | 45.4 | 14.3 |
| | | 13.7 | 10.4 | 38.5 | 18.5 |
| | | 9.8 | 9.4 | 35.5 | 17.2 |
| | | 12.2 | 11.8 | 41.9 | 14.6 |
| | **iNOS** | 106 | 88 | 265 | 125 |
| | | 109 | 95 | 310 | 131 |
| | | 98 | 106 | 289 | 94 |
| | | 89 | 89 | 239 | 115 |
| | | 115 | 108 | 198 | 97 |
| | | 83 | 92 | 227 | 136 |
| | **Caspase-3** | 87.5 | 104 | 540 | 141 |
| | | 94 | 96 | 410 | 126 |
| | | 114 | 88 | 398 | 135 |
| | | 97 | 91 | 432 | 123 |
| | | 85.5 | 93 | 591 | 152 |
| | | 122 | 83 | 425 | 189 |
| **Figure 7** | **TGF-B** | 106 | 85 | 380 | 141 |
| | | 95 | 102 | 300 | 163 |
| | | 116 | 90 | 420 | 175 |
| | | 83 | 115 | 350 | 189 |
| | | 108 | 98 | 365 | 174 |
| | | 92 | 107 | 451 | 158 |
| | **pSmad3/Smad3** | 114 | 88 | 185 | 95 |
| | | 89 | 95 | 221 | 111 |
| | | 94 | 92 | 169 | 120 |
| | | 104 | 109 | 174 | 135 |
| | | 102 | 94 | 188 | 131 |
| | | 97 | 98 | 151 | 108 |
| | **SMA** | 88 | 106 | 480 | 121 |
| | | 94 | 89 | 380 | 135 |
| | | 108 | 97 | 365 | 141 |
| | | 99 | 86 | 392 | 97 |
| | | 114 | 87 | 408 | 112 |
| | | 97 | 102 | 426 | 106 |
| **Figure 8** | **PPARg** | 118 | 121 | 41 | 79 |
| | | 104 | 115 | 29 | 85 |
| | | 85 | 135 | 45 | 81 |
| | | 93 | 122 | 35 | 71 |
| | | 105 | 98 | 28 | 82 |
| | | 95 | 116 | 36 | 78 |

EXCLI Journal 2024;23:1356-1374 – ISSN 1611-2156
Received: June 06, 2024, accepted: November 05, 2024, published: November 12, 2024

Uncropped blots
NLRP3

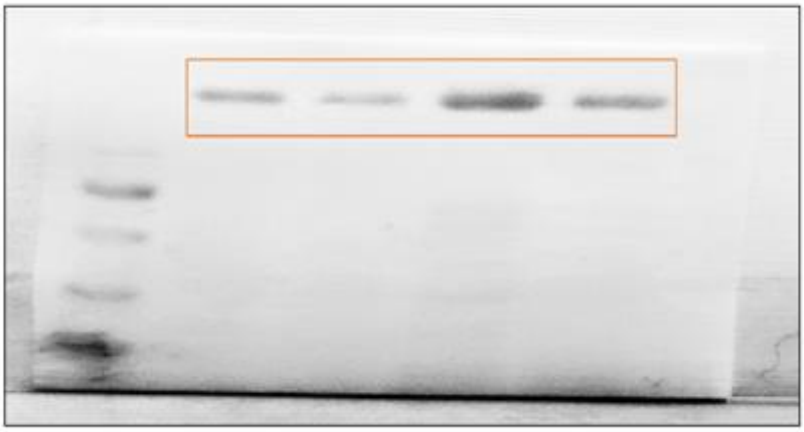

ASC

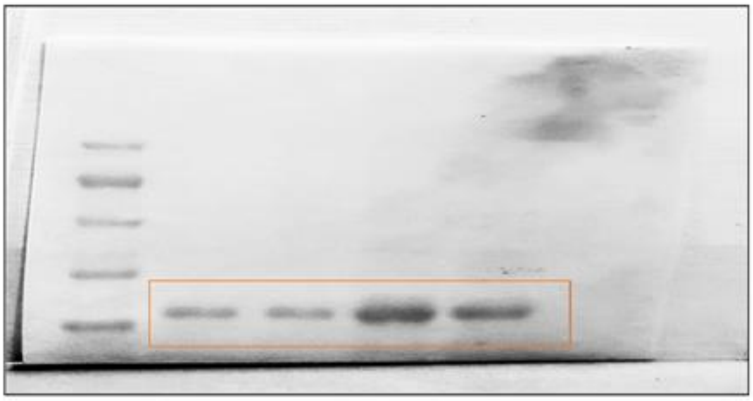

Cleaved caspase-1

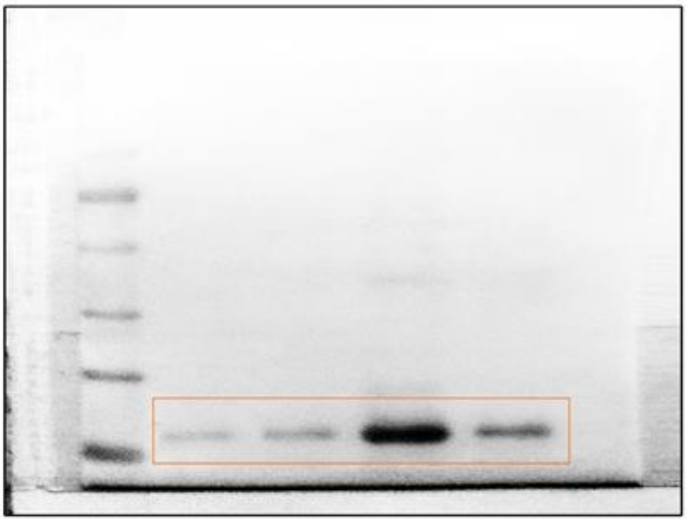

EXCLI Journal 2024;23:1356-1374 – ISSN 1611-2156
Received: June 06, 2024, accepted: November 05, 2024, published: November 12, 2024

B-actin

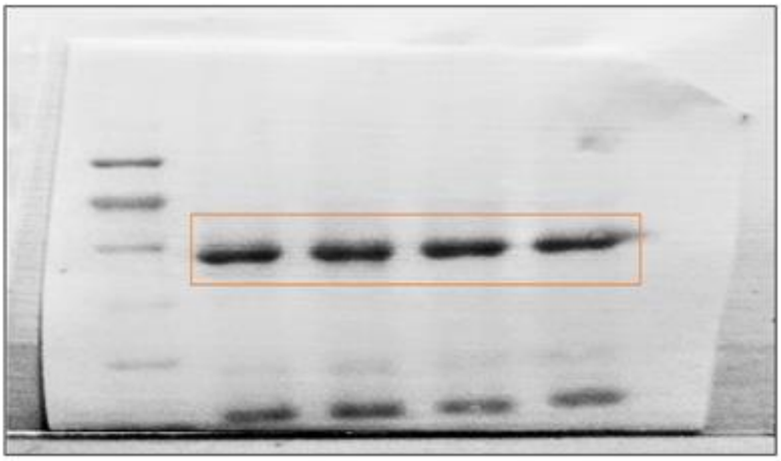

TGF-B

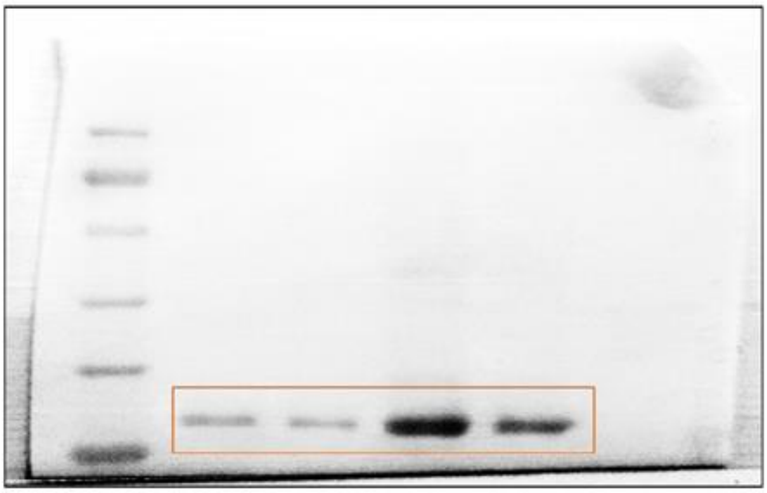

Smad3

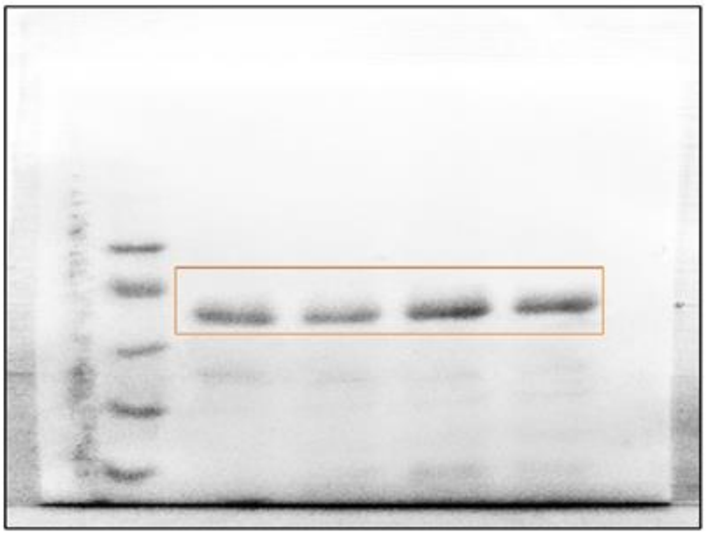

EXCLI Journal 2024;23:1356-1374 – ISSN 1611-2156
Received: June 06, 2024, accepted: November 05, 2024, published: November 12, 2024

p-Smad3

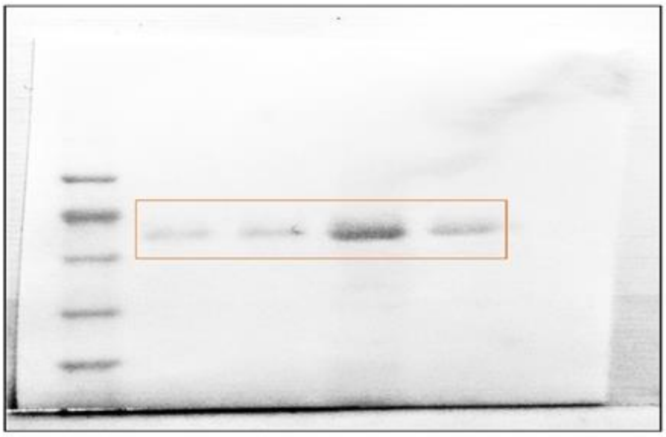

α-SMA

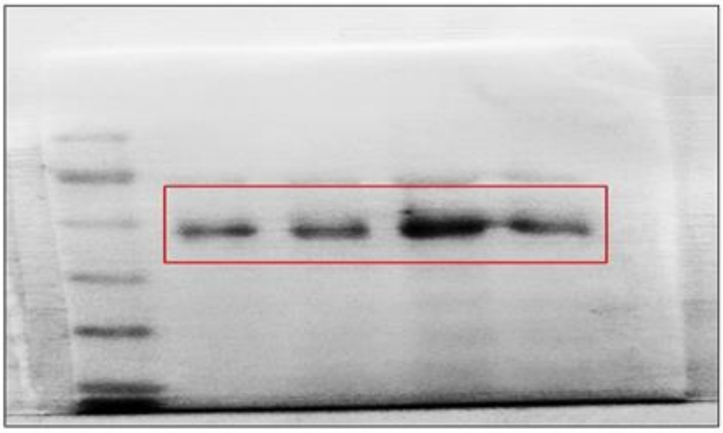

B-actin

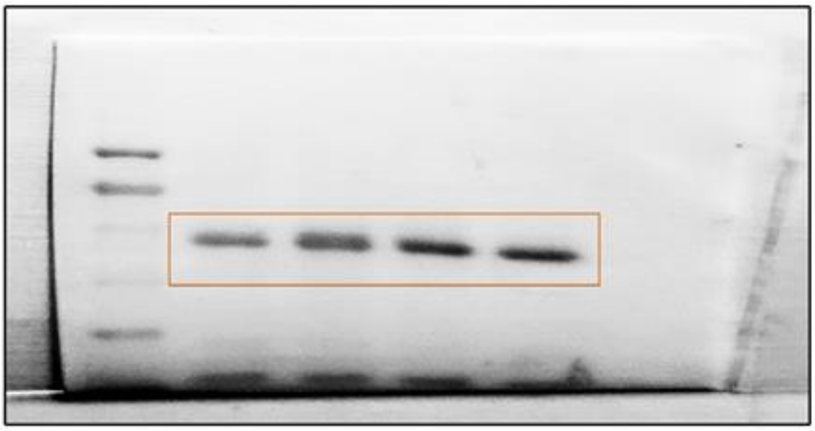

Supplement: Supplementary data [file EXCLI-23-1356-s-001.pdf]
